# Determinants of HIV-1 Late Presentation in Patients Followed in Europe

**DOI:** 10.3390/pathogens10070835

**Published:** 2021-07-02

**Authors:** Mafalda N. S. Miranda, Marta Pingarilho, Victor Pimentel, Maria do Rosário O. Martins, Anne-Mieke Vandamme, Marina Bobkova, Michael Böhm, Carole Seguin-Devaux, Roger Paredes, Rafael Rubio, Maurizio Zazzi, Francesca Incardona, Ana Abecasis

**Affiliations:** 1 Global Health and Tropical Medicine (GHTM), Institute of Hygiene and Tropical Medicine, New University of Lisbon (IHMT/UNL), 1349-008 Lisbon, Portugal; martapingarilho@ihmt.unl.pt (M.P.); victor.pimentel@ihmt.unl.pt (V.P.); mrfom@ihmt.unl.pt (M.d.R.O.M.); annemie.vandamme@uzleuven.be (A.-M.V.); ana.abecasis@ihmt.unl.pt (A.A.); 2 Laboratory Clinical and Epidemiological Virology, Department of Microbiology and Immunology, KU Leuven, Rega Institute for Medical Research, 3000 Leuven, Belgium; 3 Gamaleya Research Center of Epidemiology and Microbiology, Department of General Virology, Gamaleya Scientific Research Institute, 123098 Moscow, Russia; mrbobkova@mail.ru; 4 Department of Medicine, Saarland University Hospital, 66421 Homburg, Germany; michael.boehm@uk-koeln.de; 5 Laboratory of Retrovirology, Department of Infection and Immunity, Luxembourg Institute of Health, L-4354 Esch-sur-Alzette, Luxembourg; carole.devaux@lih.lu; 6 Infectious Diseases Department and IrsiCaixa AIDS Research Institute, Hospital Universitari Germans Trias i Pujol, 08916 Badalona, Spain; rparedes@irsicaixa.es; 7 Hospital Universitario 12 de Octubre, Universidad Complutense de Madrid, 28026 Madrid, Spain; rafaelrubiogarcia@ucm.es; 8 Department of Medical Biotechnologies, University of Siena, 53100 Siena, Italy; maurizio.zazzi@unisi.it; 9 IPRO—InformaPRO S.r.l., 98, 00152 Rome, Italy; f.incardona@informa.pro; 10 EuResist Network, 98/100, 00152 Rome, Italy

**Keywords:** HIV-1 infection, late presentation, Europe

## Abstract

To control the Human Immunodeficiency Virus (HIV) pandemic, the World Health Organization (WHO) set the 90-90-90 target to be reached by 2020. One major threat to those goals is late presentation, which is defined as an individual presenting a TCD4+ count lower than 350 cells/mm^3^ or an AIDS-defining event. The present study aims to identify determinants of late presentation in Europe based on the EuResist database with HIV-1 infected patients followed-up between 1981 and 2019. Our study includes clinical and socio-demographic information from 89851 HIV-1 infected patients. Statistical analysis was performed using RStudio and SPSS and a Bayesian network was constructed with the WEKA software to analyze the association between all variables. Among 89,851 HIV-1 infected patients included in the analysis, the median age was 33 (IQR: 27.0–41.0) years and 74.4% were males. Of those, 28,889 patients (50.4%) were late presenters. Older patients (>56), heterosexuals, patients originated from Africa and patients presenting with log VL >4.1 had a higher probability of being late presenters (*p* < 0.001). Bayesian networks indicated VL, mode of transmission, age and recentness of infection as variables that were directly associated with LP. This study highlights the major determinants associated with late presentation in Europe. This study helps to direct prevention measures for this population.

## 1. Introduction

At the end of 2019, there were 38.0 million people living with the Human Immunodeficiency Virus (HIV) and 1.7 million people were newly infected worldwide. However, 7.1 million people were still unaware of their HIV status [1].

For the control of the HIV pandemic, the World Health Organization (WHO) had set a 90-90-90 target until 2020. 90% of people living with HIV know their status, of those 90% are receiving antiretroviral therapy (ART) and of those 90% achieve viral suppression. These targets had been successful in some countries. Globally, by the end of 2019, there were 81% of people living with HIV who knew their status. Of those, 67% were receiving antiretroviral therapy and of those 59% had reached HIV viral suppression. The success of these goals is dependent on the region of origin, the vulnerability of populations and on the national HIV programs that are implemented. Yet, between 2010 and 2019, the percentage of new infections dropped by 31% [2].

New goals were set to end the pandemic by 2030, the 95-95-95 targets, based on the same definition of the previous targets. In order to attain the WHO goals by 2030, early diagnosis is essential [3].

One major concern threatening those goals is late presentation. Late presentation can have consequences in the health and treatment of infected individuals, leading to poorer outcomes and increased health care costs, since it has been shown that late presenters, especially those aged above 50 years old, are at higher risk for developing non-infectious co-morbidities and complex multimorbidity [4]. In addition, late presentation can have a negative impact on the control of the pandemic, increasing the risk of onward HIV transmission in individuals that are not aware of their HIV status. Besides, late presentation to HIV care was shown to be the main reason for virological failure [5,6].

Late presentation is defined as an individual presenting a TCD4+ count lower than 350 cells/mm^3^ or an AIDS-defining event, regardless of TCD4+ cell count. This is the definition according to the European Late Presenter Consensus working group [7]. It is estimated that Late Presenters (LP) account for 40–60% of HIV cases in Europe, in Asia the percentage of LP range from 72 to 83%, in Africa range from 35 to 89% and in Brazil, it is estimated that the percentage is near 40% [8,9,10]. For prevention and treatment of HIV, timely diagnosis and linkage to health care are essential tools [11].

The present study has the objective of identifying determinants of late presentation in Europe. To achieve this goal, we analyzed a population of patients from the EuResist database, a European database.

## 2. Results

### 2.1. Characteristics of European Population

Among 89851 HIV-1 infected patients included in the analysis, the median age was 33 (IQR: 27.0–41.0) years and 74.4% were males. From those 28889 patients (50.4%) were LP and 28388 (49.6%) were non-late presenters (NLP). The majority of patients with information about treatment status were naïve, 11487 (58.6%). 41.9% of patients were men who have sex with men (MSM) and 78.5% originated from Western Europe. The most prevalent subtype in this population was subtype B (64.4%), followed by Subtype G (20.4%), CRF 02_AG (15.9%) and Subtype A (13.5%). Most of the patients included in this study were classified as Native (75.4%) and as having Chronic Infection (59.8%) based on the ambiguity rate of the first genomic sequence. CD4 count at diagnosis and viral load at diagnosis (log10) presented a median of 348 cells/mm^3^ (IQR 170-548) and 4.4 copies/mL (IQR 3.4–5.1), respectively.

50.4% of patients were classified as LP (CD4 < 350 cells/mm^3^). Males accounted for the higher proportion of LP (74.9%). The median age of LPs was 34 years (IQR 28.0–43.0; *p* < 0.001). LPs were mainly from Western Europe and the HIV exposure category was mainly heterosexuals (77.4 and 37.1%; *p* < 0.001, respectively) (Table 1).

### 2.2. Determinants Associated with Late Presentation

In the unadjusted model (Table 2), sex was associated with LP. In the HIV exposure category, significant differences were found for MSM and Intravenous Drug Users (IDU) compared with heterosexuals. Significantly more LP were from Africa and other regions compared to Western Europe. In addition, the variables age at diagnosis, viral load, subtype, recentness of infection and migrant status were significantly associated with LP.

Determinants associated with late presentation were age at diagnosis (Table 2): patients with less than 30yo had lower probability of being late presenters and patients aged above 56yo had higher probability of being late presenters when compared with patients aged between 31 and 55yo (>18yo: aOR 0.31 (0.20–0.49), *p* < 0.001; 19–30yo: aOR 0.46 (0.34–0.62), *p* < 0.001; >56: aOR 1.70 (1.49–1.94), *p* = 0.004), transmission via MSM had lower probability when compared with heterosexuals (aOR 0.74 (0.64–0.86); *p* < 0.001). Patients originating from Africa and South America had 1.76 and 1.41 more probability, respectively, of presenting late than those from Western Europe (aOR 1.76 (1.37–2.26), *p* < 0.001; aOR 1.41 (1.07–1.87), *p* = 0.015, respectively) and patients presenting with a viral load between 4.1 and 5.0 and higher than 5.1 had a higher probability of being LP than those with a viral load lower than 4.0 (aOR 1.45 (1.37–1.53) and aOR 3.41 (3.21–3.62); *p* < 0.001 and *p* < 0.001, respectively). As expected, but confirming the reliability of our classification of recentness of infection based on the ambiguity rate, patients with a recent infection—as classified based on the ambiguity rate of the genomic sequence from the first drug resistance test—had a lower probability of being LP than those classified as being chronically infected (aOR 0.61 (0.55–0.68); *p* < 0.001).

### 2.3. Bayesian Network

**Figure 1:**
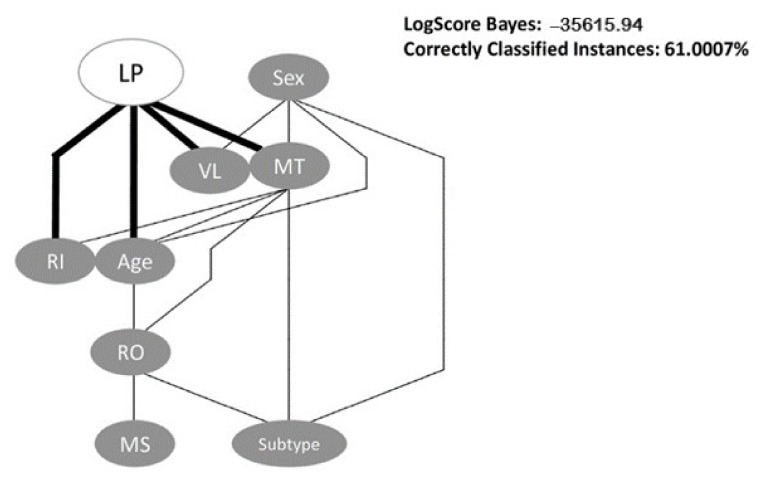
Bayesian Network analysis of association between variables investigated in the study. The BayesNet classifier and the HillClimber algorithm were used as implemented in the WEKA software. LP—Late Presenters; VL—Viral Load; MT—Mode of Transmission; RI—Recentness of infection; RO—Region of Origin; MS—Migrant Status.

For the bayesian network, we used the HillClimber algorithm with nine as the maximum number of parents a node in BN can have. This algorithm is based on a “hill climbing adding and deleting arcs with no fixed ordering of variables” [12]. The BN had a LogScore Bayes of −35615.94 and an accuracy of 61%. In the BN (Figure 1), LPs are directly associated with the viral load, recentness of infection, mode of transmission and age, as we can see in the figure below, those were direct links between the nodes. The indirectly associated links were between LP and region of origin. As we can also see in the figure, there was no direct link between those two nodes. We can see that the mode of transmission is the variable with more direct associations and the variable sex is the only one that is not associated with LP. This BN is in accordance with our logistic regression model.

The variables Subtype and Migrant status had been removed from the logistic regression model due to the conflict with the variable region of origin. As we can see in Figure 1, the region of origin is directly associated with those two variables and that the migrant status is only associated with region of origin.

### 2.4. Ambiguity Rate and CD4 Analysis

We performed an analysis to understand the association between CD4 count and the ambiguity rate overall and on subtype B, non-B and G. This association was inversely proportional in all correlations, this means that for higher values of CD4 count the ambiguity rate is lower. In this study, the LP population had higher ambiguity rates in their sequences, since their CD4 count is lower. We also performed a linear regression in order to explain how much of the CD4 count could the ambiguity rate explain. We divided that analysis in the same categories as mentioned above and the higher result was from only individuals with non-B subtype, in which the ambiguity rate explained 5% of the variation from CD4 count (Table A1, Table A2, Table A3 and Table A4).

### 2.5. Analysis of Late Presenters Rate over Time

**Figure 2:**
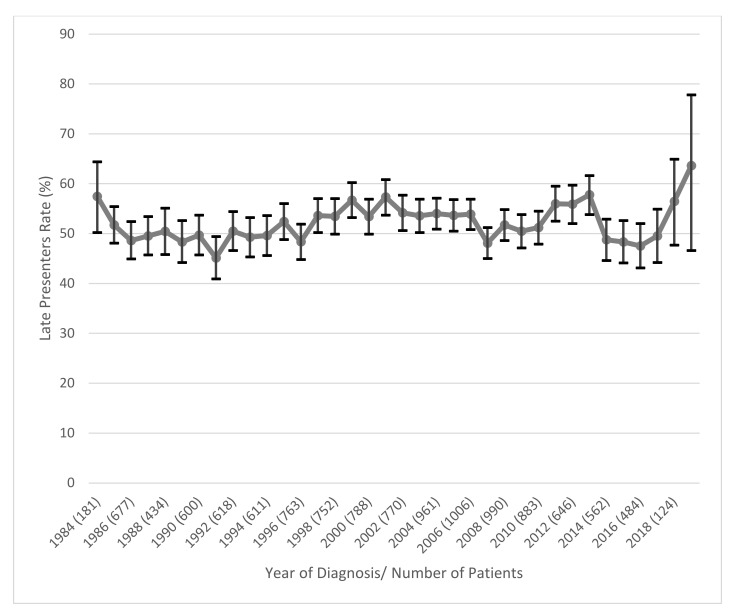
Evolution of Late Presenters rate per Year. Vertical black lines represent confidence intervals.

We also constructed a graph to evaluate the evolution in time of the rate of LP (Figure 2). The confidence intervals were also calculated for each point. We did not include in the analysis the first three years (1981–1983) since the total number of patients in those years was low and the confidence intervals had high values. In 2019 the sample size was also small, but we included this year in the analysis to see the trend that LPs in Europe will have. As we can see in the graph, LPs have had constant values through the years. In 1984 we had 57.5% LPs, in 1991 we had the lowest value of LPs (45.1%). The evolution through the years maintained between 45 and 60% the rate of LP. Since 2017 the rate of LPs was growing until 2019 that peaked, beyond 60%.

## 3. Discussion

This study had the goal of explaining the determinants of late presentation for HIV-1 infection in Europe.

In our population, late presenters represented 50.4% of the patients. A study in Georgia, using the same definition of late presentation as we used, reported 63.4% of late presenters. Another study analyzing late presentation in different settings indicated a rate of late presentation ranging between 40 and 67%, depending on the region of study. This study corresponded to the Swiss data incorporated in the COHERE study, a Collaboration of Observational HIV Epidemiological Research Europe Study. Our results are concordant with the results reported in these studies [13,14,15].

In our study, late presenters were more frequently males, with heterosexual transmission, from Western Europe and aged between 31 and 55 years old. In a study in East of England, the percentage of late presenters was higher in older patients and patients with heterosexual contact, when compared with homosexual and bisexual contact. Furthermore, according to other studies in Poland and the Netherlands, males were also more prevalent in the late presenters’ population. These results are consistent with our study [16,17,18].

Patients originated from Africa had a higher probability of being LPs when compared to patients originated from Western Europe. This percentage of African migrants in the LP population can be explained by the lower access to health care. Furthermore, African migrants have a higher probability of being in conditions of unemployment, poverty and poorer household, which further increase their barriers to access to health care. A positive status for HIV also stigmatizes individuals, and they fear the reactions of their communities, since HIV is mostly associated among these communities with inappropriate and promiscuous sexual behavior [19]. The migrants of our study from South America were mainly from Brazil and the LP rate was lower than the NLPs. This can be explained in two ways: Brazil has a concentrated HIV epidemic among MSM population and that population is frequently tested [20]. These results are in accordance with HIV studies about the migrant population [21,22,23].

The results from a previous study showed a statistically significant correlation between late presentation and IDUs [24]. In our study, we found this significant association between LP and IDU in our univariate analysis, but in the logistic regression analysis, we only found significant the association between MSM when compared to heterosexuals. The prevalence of HIV-positive IDU population is mainly from Eastern Europe. In our study, the IDU group maybe underrepresented since the larger proportion of cases are from Western Europe, in which the major mode of transmission is through heterosexual and MSM contacts [25,26].

We also studied the association between CD4 count and the ambiguity rate of the sequences included in this study. Our results show a negative correlation between CD4 count and the ambiguity rate, for lower values of CD4 we had higher values of the ambiguity rate. There is still little information regarding this topic, but our results were in accordance with a study about sequence ambiguity and HIV incidence trends [27]. In fact, the ambiguity rate could be an alternative variable to be used for the definition of Late Presentation. As we know, the initial drop of CD4 count in the acute phase of HIV infection can be a cause of bias when we define Late Presentation based on a CD4 count lower than 350 cells/mm^3^.

The results from the graph showed stable and high values for LPs rate. This indicates that LPs were and remain a big part of the HIV epidemic and represent a major threat to treatment and prevention strategies.

**Figure 3:**
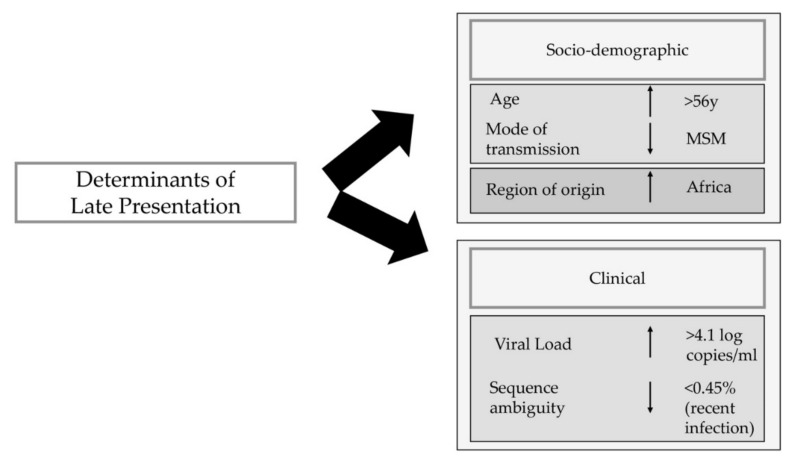
Schematic representation of determinants of late presentation based on logistic regression and Bayesian networks analysis. The light grey box indicates the direct determinants, and the dark grey box indicates the indirect determinants of late presentation. Individuals older than 56yo and originated from Africa had higher probability of being late presenters, Men who have sex with men were less likely to be late presenters. In the clinical determinants there were only direct associated determinants. Individuals with a viral load higher than 4.1 copies/mL had higher probability of being late presenters and individuals with a lower rate of sequence ambiguity had lower probability of being late presenters. MSM-Men who have sex with Men.

The main goal of this study was to identify determinants associated with late presentation. Those determinants included age at diagnosis, mode of transmission, region of origin, recentness of infection and viral load at diagnosis (Figure 3). Our results were in concordance with other previously published studies [13,28,29].

The last study about late presentation in Europe was published in 2015 and the timeline of the study was between 2010 and 2013. This was an update from the first study published in 2013, with a timeline of analysis between 2000 and 2011 [29,30]. Our study analyzes a European database with a timeline between 1981 and 2019. The main strength of our study was the database used, which is one of the largest datasets and integrates clinical, socio-demographic and viral genotypic information from HIV-1 patients from all over Europe. This large dataset allows for a robust analysis of the data, and up to date information regarding late presentation. In addition, we can analyze trends in the evolution of late presentation in Europe.

The major limitation of our study was the lack of information about the stage of HIV infection and AIDS-defining events. While we used the ambiguity rate to minimize this problem, we only used the definition of a CD4 count below 350 cells/mm^3^ to define an individual as LP or NLP.

Yet, this study is the most recent update on the HIV epidemic of late presentation in Europe, since the last one was published in 2015.

Since late presentation is a major obstacle to the 95-95-95 targets, it is necessary to reinforce the follow-up of this population. Increased HIV testing is key to reduce late presentation since it results in earlier HIV diagnosis. Prevention measures like targeting the vulnerable populations and increasing screening programs for those populations are the most urgent strategies to halt and decrease the percentage of late presenters. In low- and middle-income countries, point-of-care testing would be a major advance to stop the spread of the virus by those who do not know their serological status and therefore decreasing late presentation at diagnosis.

## 4. Materials and Methods

### 4.1. Study Group

Our study includes clinical and socio-demographic information from 89851 HIV-1 infected patients from the EuResist Integrated Database (EIDB) between 1981 and 2019. The EuResist integrated database (EIDB) is one of the largest existing datasets which integrate clinical, socio-demographic and viral genotypic information from HIV-1 patients. It integrates longitudinal, periodically updated data mainly from Italy (ARCA database), Germany (AREVIR database) Spain (CoRIS and IRISCAIXA), Sweden, Belgium, Portugal and Luxembourg [31,32,33].

In this study, information from the ARCA, AREVIR, Luxembourg, IRISCAIXA, Portugal, Russia, United Kingdom and CoRIS databases were used.

### 4.2. Subtyping

The genomic data included HIV-1 protease and reverse transcriptase sequences, generated through routine drug resistance testing and as stored in the EuResist database. Only the first HIV genomic sequence per patient was considered.

HIV-1 subtyping was performed using the consensus of the result obtained through three different tools: Rega HIV Subtyping Tool (https://www.genomedetective.com/app/typingtool/hiv, accessed on 1 July 2021) [34], COMET: adaptive context-based modeling for HIV-1 (https://comet.lih.lu, accessed on 1 July 2021) [35] and SCUEAL (http://classic.datamonkey.org/dataupload_scueal.php, accessed on 1 July 2021).

### 4.3. Study Variables

We used the information from the EuResist database regarding the following variables: Country of follow-up, Year of Birth, Gender, Country of Origin, Mode of transmission, Date of the first HIV Positive test, Date and value of the first CD4 count, Date and value of the first Viral Load count, first genomic sequence and sample collection date, Date of therapy initiation.

With this information we created new variables such as:

- Migrant/Native-Based on Country of Origin and Country of Follow-up (if country of origin and country of follow-up is the same, then patient is native; if country of origin and country of follow-up is not the same, then patient is migrant)
- Age at Diagnosis-Based on the difference between Year of Birth and Date of the first HIV Positive test;
- Region of Origin- Based on Country of Origin;
- Treatment Status at date of first CD4 count-Based on the difference between sample collection date of first CD4 count and first therapy date; for purposes of classification of Late Presentation, only patients naïve at date of first CD4 count were considered;
- Treatment Status at date of first Drug Resistance Test-based on the difference between sample collection date for first drug resistance test and date of start of first therapy;

After creating these two variables, for quality control purposes, we only included in the analysis patients for which treatment status at date of first CD4 count and Treatment Status at date of first Drug Resistance test were consistent.

- Recentness of infection-Based on ambiguity rate of genomic sequences. We defined Chronic infection as an ambiguity value higher than 0.45% and Recent infection as an ambiguity value equal or below 0.45% [36]. Additionally, only genomic sequences larger than 500 nucleotides and with ambiguity rate lower than 2.5% were considered.
- LP vs. NLP- Based on CD4 count, LP were defined as patients with CD4 count lower than 350 cells/mm^3^ and NLP were defined as patients with CD4 count higher than 350 cells/mm^3^.

### 4.4. Statistical Analysis

The proportion and median (interquartile range, IQR) of LP and non- Late presenters (NLP) were calculated for every categorical and continuous variable, respectively. Our interest variables were compared with the categorical variables with Chi-square test, and continuous variables with Mann–Whitney U test.

To study the relationship between our dependent variable (LP or NLP) and the independent variables, logistic regression models were calculated. We first presented the logistic regression with the unadjusted odds ratios (uOR) and confidence intervals at 95% (95% CI), in order to see the probability of our event, the dependent variable, (late presentation vs non-late presentation) on the occurrence of each independent variable, individually, e.g., the probability of a woman being late presenter. Variables with a *p*-value < 0.05 were considered to enter the model since it is the most used threshold. The final model for LP vs. NLP was adjusted for sex, this variable was forced into the model regardless of its significance and the reference class was women. The final model included only the variables that were considered statistically significant (*p* < 0.05) and the variables that suited the best regression model according to the backward stepwise regression analysis through SPSS. The odds ratio and 95% confidence intervals were calculated for those variables as well. Data were analyzed using RStudio (Version 1.2.5033) and SPSS (Version 26.0.0.0).

### 4.5. Bayesian Networks

A Bayesian network (BN) is a tool that consists of a directed acyclic graph (DAG), made of nodes and directed links between the nodes, which allows us to understand the representation of a probabilistic distribution. Each node is a representation of a variable, and the links indicate that one node is directly influencing another. The lack of a direct link does not mean that one variable is not associated with another. These networks are able to intuitively create causal links between variables since they are built from probability distributions and for prediction [37].

We constructed a BN to analyze the association between all variables, specifically, we wanted to see how the variables were associated with one another. With the different levels and connections between variables, it is possible to see if they are directly or indirectly associated. We used the WEKA software version 3.8.5. WEKA stands for Waikato Environment for Knowledge Analysis. After the upload of the dataset, the first step is to choose a classifier to start the analysis. We used a statistical-based learning scheme, the Bayes classifier, specifically the BayesNet [38]. After choosing the classifier, we used different search algorithms as a local score structure learning. Our final choice of algorithm was based on the LogScore Bayes value and the percentage of correctly classified instances.

## 5. Conclusions

In summary, late presentation still accounts for 50% of the new diagnosis in Europe. Its most important determinants are age at diagnosis, mode of transmission, region of origin and viral load at diagnosis (Figure 3). In addition, the evolution of the rate of late presentation through the years was stable, except for the last two years analyzed (2018 and 2019) when that rate showed an increase. This study highlights the major determinants associated with late presenters in Europe, and this will help to strengthen some prevention measures.

## Figures and Tables

**Table 1:** Demographic and patient characteristics. Other in mode of transmission includes blood transfusions and vertical transmission. Other in region of origin and infection includes North and Central America, Asian and Oceania continents. *p*-values retrieved with chi-square test and Mann–Whitney U test.

Patient Characteristics	Total	Late Presenters	Non-Late Presenters	*p*-Value
Total, *n* (%)	89,851 (100%)	28,889 (50.4%)	28,388 (49.6%)	
Sex, *n* (%)	81,777 (91.0%)	27,972 (50.6%)	27,315 (49.4%)	<0.001
Male	60,852 (74.4%)	20,955 (74.9%)	20,969 (76.8%)	
Female	20,925 (25.6%)	7017 (25.1%)	6346 (23.2%)	
Treatment status, *n* (%)	19,605 (21.8%)	10,905 (55.6%)	8700 (44.4%)	<0.001
Naïve	11,487(58.6%)	6040 (55.4%)	5447 (62.6%)	
Treated	8118 (41.4%)	4865 (44.6%)	3253 (37.4%)	
Median age at diagnosis in years IQR, *n* (%)	25,530 (28.4%)	11,929 (52.3%)	10,897 (47.7%)	<0.001
	33.0 (27.0–41.0)	34.0 (28.0–43.0)	31.0 (26.0–39.0)	
≤18	700 (2.7%)	241 (2.0%)	340 (3.1%)	<0.001
19–30	9767 (38.3%)	4002 (33.5%)	4823 (44.3%)	
31–55	13,815 (54.1%)	6920 (58.0%)	5384 (49.4%)	
≥56	1248 (4.9%)	766 (6.4%)	350 (3.2%)	
Mode of transmission, *n* (%)	47,007 (52.3%)	21,283 (49.5%)	21,677 (50.5%)	<0.001
Heterosexual	15,165 (32.3%)	7894 (37.1%)	6071 (28.0%)	
MSM	19,696 (41.9%)	7657 (36.0%)	10,693 (49.3%)	
IDU	9532 (20.3%)	4453 (20.9%)	3896 (18.0%)	
Other	2614 (5.6%)	1279 (6.0%)	1017 (4.7%)	
Region of origin, *n* (%)	54,529 (60.7%)	21,584 (50.1%)	21,495 (49.9%)	<0.001
Western Europe	42,790 (78.5%)	16,693 (77.4%)	17,398 (81.0%)	
Eastern Europe	1862 (3.4%)	693 (3.2%)	672 (3.1%)	
Africa	5349 (9.8%)	2250 (10.4%)	1422 (6.6%)	
South America	3233 (5.9%)	1341 (6.2%)	1460 (6.8%)	
Other	1286 (2.4%)	607 (2.8%)	543 (2.5%)	
Subtype, *n* (%)	54,176 (60.3%)	17,449 (52.7%)	15,638 (47.3%)	<0.001
HIV-1 Subtype B	35,454 (64.4%)	11,966 (68.6%)	11,745 (75.1%)	
HIV-1 Subtype non-B	18,722 (34.6%)	5483 (31.4%)	3893 (24.9%)	
Distribution of non-B Subtypes				
HIV-1 CRF 01_AE	447 (2.4%)	183 (3.3%)	108 (2.8%)	
HIV-1 CRF 02_AG	2973 (15.9%)	871 (15.9%)	556 (14.3%)	
HIV-1 CRF 06_cpx	248 (1.3%)	81 (1.5%)	58 (1.5%)	
HIV-1 CRF 14_BG	1106 (5.9%)	337 (6.1%)	203 (5.2%)	
HIV-1 Subtype A	2521 (13.5%)	626 (11.4%)	527 (13.5%)	
HIV-1 Subtype C	1943 (10.4%)	550 (10.0%)	400 (10.3%)	
HIV-1 Subtype D	307 (1.6%)	102 (1.9%)	74 (1.9%)	
HIV-1 Subtype F	1619 (8.6%)	444 (8.1%)	362 (9.3%)	
HIV-1 Subtype G	3815 (20.4%)	1156 (21.1%)	701 (28.0%)	
Others	3743 (20.0%)	1133 (20.7%)	3893 (23.2%)	
Migrant status, *n* (%)	54,520 (60.7%)	21,584 (50.1%)	21,495 (49.9%)	<0.001
Migrant	13,408 (24.6%)	5588 (25.9%)	4895 (22.8%)	
Native	41,112 (75.4%)	15,996 (74.1%)	16,600 (77.2%)	
Recentness of infection, *n* (%)	50,132 (55.8%)	15,897 (52.6%)	14,304 (47.4%)	<0.001
Chronic	29,972 (59.8%)	11,069 (69.6%)	7803 (54.6%)	
Recent	20,160 (40.2%)	4828 (30.4%)	6501 (45.4%)	
Median CD4 count at diagnosis (cells/mL) IQR, *n* (%)	57,277 (63.7%)	28,889 (50.4%)	28,388 (49.6%)	<0.001
	348.0 (170.0–548.0)	172.0 (69.0–264.0)	550.0 (442.0–720.0)	
Viral Load at diagnosis (log10 copies/mL) IQR, n (%)	34,046 (37.9%)	15,106 (50.8%)	14,605 (49.2%)	<0.001
	4.4 (3.4–5.1)	4.7 (3.8–5.3)	4.1 (3.1–4.8)	
≤4.0	12,994 (38.2%)	4485 (29.7%)	6819 (46.7%)	<0.001
4.1–5.0	11,715 (34.4%)	5034 (33.3%)	5295 (36.3%)	
≥5.1	9337 (27.4%)	5587 (37.0%)	2491 (17.1%)	

**Table 2:** Logistic Regression for determinants associated with late presentation. Ref—Reference category; aOR-adjusted Odds Ratio; Other in mode of transmission include transfusions; Other in region of origin include Latin and North American Countries; MSM- Men have sex with men; IDU- Injection drug users.

Late Presenters/ Non-Late Presenters	Unadjusted		Final Model	
Variable	OR (95%CI)	*p*-Value	aOR (95%CI)	*p*-Value
Sex				
Female	Ref	Ref	Ref	Ref
Male	0.90 (0.87–0.94)	<0.001	1.05 (0.91–1.21)	0.522
Age at diagnosis	1.03 (1.02–1.03)	<0.001		
Age groups				
<18	0.55 (0.47–0.65)	<0.001	0.48 (0.33–0.69)	<0.001
19–30	0.65 (0.61–0.68)	<0.001	0.70 (0.63–0.79)	<0.001
31–55	Ref	Ref	Ref	Ref
>56	1.70 (1.49–1.94)	<0.001	1.54 (1.15–2.06)	0.004
Mode of transmission				
Heterosexual	Ref	Ref	Ref	Ref
MSM	0.55 (0.53–0.58)	<0.001	0.74 (0.64–0.86)	<0.001
IDU	0.88 (0.83–0.93)	<0.001	1.12 (0.96–1.31)	0.137
Other	0.97 (0.88–1.06)	0.462	1.29 (0.99–1.70)	0.062
Region of Origin				
Western Europe	Ref	Ref	Ref	Ref
Eastern Europe	1.08 (0.97–1.20)	0.191	1.07 (0.78–1.48)	0.683
Africa	1.65 (1.54–1.77)	<0.001	1.76 (1.37–2.26)	<0.001
South America	0.96 (0.89–1.03)	0.267	1.41 (1.07–1.87)	0.015
Other	1.17 (1.04–1.31)	0.011	1.39 (0.92–2.09)	0.118
Subtype				
HIV-1 Subtype B	Ref	Ref		
HIV-1 Subtype non-B	1.38 (1.32–1.45)	<0.001		
Migrant Status				
Migrant	Ref	Ref		
Native	0.84 (0.81–0.88)	<0.001		
Recentness of infection				
Chronic	Ref	Ref	Ref	Ref
Recent	0.52 (0.50–0.55)	<0.001	0.61 (0.55–0.68)	<0.001
Log Viral load at diagnosis	1.45 (1.42–1.48)	<0.001		
Log Viral load groups				
<4.0	Ref	Ref	Ref	Ref
4.1–5.0	1.45 (1.37–1.53)	<0.001	1.37 (1.22–1.55)	<0.001
>5.1	3.41 (3.21–3.62)	<0.001	3.41 (2.96–3.91)	<0.001

## Data Availability

Restrictions apply to the availability of these data. Data was obtained from the EuResist Network and is available for request through a study application form at https://www.euresist.org/become-a-partner with the permission of the EuResist Network.

## Appendix A

**Table A1:** Linear Regression for CD4 count vs. Ambiguity rate.

CD4 Count	R^2^	Unstandardized B	Standardized Coefficients Beta	*p*-Value	95% Confidence Interval for B		Spearman’s Correlation	
					Lower Bound	Upper Bond	*p*-Value	Correlation Coefficient
**Ambiguity Rate**	0.023	−69.52	−0.152	<0.001	−74.61	−64.44	<0.001	−0.190

**Table A2:** Linear Regression for CD4 count vs. Ambiguity rate for Subtype B.

CD4 Count	R^2^	Unstandardized B	Standardized Coefficients Beta	*p*-Value	95% Confidence Interval for B		Spearman’s Correlation	
					Lower Bound	Upper Bond	*p*-Value	Correlation Coefficient
**Ambiguity Rate**	0.015	−57.11	−0.123	<0.001	−63.27	−50.95	<0.001	−0.159

**Table A3:** Linear Regression for CD4 count vs. Ambiguity rate for Subtype non-B.

CD4 Count	R^2^	Unstandardized B	Standardized Coefficients Beta	*p*-Value	95% Confidence Interval for B		Spearman’s Correlation	
					Lower Bound	Upper Bond	*p*-Value	Correlation Coefficient
**Ambiguity Rate**	0.051	−96.72	−0.226	<0.001	−105.54	−87.91	<0.001	−0.265

**Table A4:** Linear Regression for CD4 count vs. Ambiguity rate for Subtype G.

CD4 Count	R^2^	Unstandardized B	Standardized Coefficients Beta	*p*-Value	95% Confidence Interval for B		Spearman’s Correlation	
					Lower Bound	Upper Bond	*p*-Value	Correlation Coefficient
**Ambiguity Rate**	0.014	−52.78	−0.120	<0.001	−73.38	−32.17	<0.001	−0.144
